# Evaluation of clinical practice guidelines using the AGREE instrument: comparison between data obtained from AGREE I and AGREE II

**DOI:** 10.1186/s13104-017-3041-7

**Published:** 2017-12-08

**Authors:** Kanako Seto, Kunichika Matsumoto, Takefumi Kitazawa, Shigeru Fujita, Shimpei Hanaoka, Tomonori Hasegawa

**Affiliations:** Department of Social Medicine, School of Medicine, Toho University, Tokyo, Japan

**Keywords:** Clinical practice guidelines, AGREE (Appraisal of Guidelines for Research and Evaluation) instrument, Data transfer, Data mapping

## Abstract

**Objective:**

The Appraisal of Guidelines for Research and Evaluation (AGREE) is a representative, quantitative evaluation tool for evidence-based clinical practice guidelines (CPGs). Recently, AGREE was revised (AGREE II). The continuity of evaluation data obtained from the original version (AGREE I) has not yet been demonstrated. The present study investigated the relationship between data obtained from AGREE I and AGREE II to evaluate the continuity between the two measurement tools.

**Results:**

An evaluation team consisting of three trained librarians evaluated 68 CPGs issued in 2011–2012 in Japan using AGREE I and AGREE II. The correlation coefficients for the six domains were: (1) scope and purpose 0.758; (2) stakeholder involvement 0.708; (3) rigor of development 0.982; (4) clarity of presentation 0.702; (5) applicability 0.919; and (6) editorial independence 0.971. The item “Overall Guideline Assessment” was newly introduced in AGREE II. This global item had a correlation coefficient of 0.628 using the six AGREE I domains, and 0.685 using the 23 items. Our results suggest that data obtained from AGREE I can be transferred to AGREE II, and the “Overall Guideline Assessment” data can be determined with high reliability using a standardized score of the 23 items.

**Electronic supplementary material:**

The online version of this article (10.1186/s13104-017-3041-7) contains supplementary material, which is available to authorized users.

## Introduction

Clinical practice guidelines (CPGs) are “statements that include recommendations intended to optimize patient care that are informed by a systematic review of evidence and an assessment of the benefits and harms of alternative care options.” [1]. CPGs are a representative tool for standardizing medical interventions and improve healthcare quality. In Japan, CPG development, using evidence-based medicine (EBM), began in the late 1990s with government support. Currently, 30–40 CPGs are developed per year, mainly by academic societies.

With the spread of CPGs in Japan, infrastructure to promote their use is also being developed. This includes clearing houses and standard manuals for developing CPGs. The Toho University Medical Media Center and the Medical Information Network Distribution Service Guideline Center of the Japan Council for Quality Health Care both operate CPG clearing houses [2, 3].

The Appraisal of Guidelines for Research and Evaluation (AGREE) instrument, developed by the AGREE Enterprise, is a quantitative method for evaluating CPGs. The AGREE instrument determines items that must be satisfied by CPGs, and is expected to facilitate cost effective CPG development and improve CPG quality [4]. In 2010, the original version (AGREE I) was revised and published as AGREE II [5–7]. Several studies evaluated CPGs using the AGREE I or AGREE II [8–10]. However, the continuity of the data obtained from AGREE I and AGREE II has not yet been demonstrated. The AGREE I was widely used and there is large amount of associated data; investigation of the continuity and conversion of data between AGREE I and II is necessary to make full use of AGREE I data.

We investigated the continuity of AGREE I and AGREE II data, and the conversion method from AGREE I data to AGREE II data.

## Main text

### Methods

A team consisting of three experienced librarians evaluated 68 CPGs, based on EBM issued in 2011–2012 using the AGREE I [11] and AGREE II [12]. The evaluated CPGs were all issued in 2011–2012 in Japan. Their contents were checked and judged by expert librarians as to whether they were prepared using EBM methodology, or not. The librarians who evaluated the CPGs have knowledge about the CPG preparation and experience using the AGREE tool. The librarians conducted independent evaluations and did not adjust the result; the results were aggregated into standardized scores. Correlation coefficients were calculated for the domains and items of the two instruments.

AGREE I comprised one overall assessment item and six domains: (1) scope and purpose; (2) stakeholder involvement; (3) rigor of development; (4) clarity of presentation; (5) applicability; (6) editorial independence, totaling 23 items. Each item is rated on a 4-point Likert scale (1 = “Strongly Disagree” to 4 = “Strongly Agree”). A standardized score for each domain was calculated according the formula shown below:

$$\left[ {{{\left( {{\text{obtained score}} - {\text{minimum possible score}}} \right)} \mathord{\left/ {\vphantom {{\left( {{\text{obtained score}} - {\text{minimum possible score}}} \right)} {\left( {{\text{maximum possible score}} - {\text{minimum possible score}}} \right)}}} \right. \kern-0pt} {\left( {{\text{maximum possible score}} - {\text{minimum possible score}}} \right)}}} \right] \times 100\% .$$

For example, the scope and purpose domain consists of three items; the sum of the maximum possible score is 3 × 3 × 3 = 27, and the sum of the minimum possible score is 1 × 3 × 3 = 9 [11].

AGREE II is based on AGREE I, incorporating four distinct changes. First, the rating scale was changed from a 4-point to a 7-point Likert scale (1 = “Strongly Disagree” to 7 = “Strongly Agree”). Second, an item was added as a second overall guideline assessment item: “Rate the overall quality of this guideline”. Third, the wording or expression of several items was changed, although the meaning of the items was preserved. Finally, Q7 (AGREE I) “The guideline has been piloted among end users” was removed, and was incorporated in Q19 (AGREE II) “The guideline describes facilitators and barriers to its application” and a new item Q9 (AGREE II) “The strengths and limitations of the body of evidence are clearly described”. Therefore, Q7 (AGREE I) and Q9 (AGREE II) were excluded from analysis in the present study. A comparison of AGREE I and AGREE II items is shown in Table 1.

**Table 1:** Comparison between the AGREE I and AGREE II

AGREE I			AGREE II			Change from AGREE I to AGREE II
Domain	No	Item	Item	No	Domain	
1. Scope and purpose	1	The overall objective(s) of the guideline is (are) specifically described	The overall objective(s) of the guideline is (are) specifically described	1	1. Scope and purpose	No change
	2	The clinical question(s) covered by the guideline is (are) specifically described	The health question(s) covered by the guideline is (are) specifically described	2		Change in underline part
	3	The patients to whom the guideline is meant to apply are specifically described	The population (patients, public, etc.) to whom the guideline is meant to apply is specifically described	3		Change in underline part
2. Stakeholder involvement	4	The guideline development group includes individuals from all the relevant professional groups	The guideline development group includes individuals from all the relevant professional groups	4	2. Stakeholder involvement	No change
	5	The patients’ views and preferences have been sought	The views and preferences of the target population (patients, public, etc.) have been sought	5		Change in underline part
	6	The target users of the guideline are clearly defined. No	The target users of the guideline are clearly defined. No	6		No change
	7	The guideline has been piloted among end users				Delete item. Incorporated into user guide description of item 19
3. Rigour of development	8	Systematic methods were used to search for evidence	Systematic methods were used to search for evidence	7	3. Rigour of development	No change, renumber to 7
	9	The criteria for selecting the evidence are clearly described	The criteria for selecting the evidence are clearly described	8		No change, renumber to 8
			The strengths and limitations of the body of evidence are clearly described	9		New item
	10	The methods for formulating the recommendations are clearly described	The methods for formulating the recommendations are clearly described	10		No change
	11	The health benefits, side effects, and risks have been considered in formulating the recommendations	The health benefits, side effects, and risks have been considered in formulating the recommendations	11		No change
	12	There is an explicit link between the recommendations and the supporting evidence	There is an explicit link between the recommendations and the supporting evidence	12		No change
	13	The guideline has been externally reviewed by experts prior to its publication	The guideline has been externally reviewed by experts prior to its publication	13		No change
	14	A procedure for updating the guideline is provided	A procedure for updating the guideline is provided	14		No change
4. Clarity of presentation	15	The recommendations are specific and unambiguous	The recommendations are specific and unambiguous	15	4. Clarity of presentation	No change
	16	The different options for management of the condition are clearly presented	The different options for management of the condition or health issue are clearly presented	16		Change in underline part
	17	Key recommendations are easily identifiable	Key recommendations are easily identifiable	17		No change
	18	The guideline is supported with tools for application	The guideline describes facilitators and barriers to its application	19	5. Applicability	Change in underline part, renumber to 19, change in domain from #4 clarity of presentation to #5 applicability
5. Applicability	19	The potential organizational barriers in applying the recommendations have been discussed	The guideline provides advice and/or tools on how the recommendations can be put into practice	18		Change in underline part, renumber to 18.
	20	The potential cost implications of applying the recommendations have been considered	The potential resource implications of applying the recommendations have been considered	20		Change in underline part
	21	The guideline presents key review criteria for monitoring and/ or audit purposes	The guideline presents monitoring and/ or auditing criteria	21		Change in underline part
6. Editorial independence	22	The guideline is editorially independent from the funding body	The views of the funding body have not influenced the content of the guideline	22	6. Editorial independence	Change in underline part
	23	Conflicts of interest of guideline development members have been recorded	Competing interests of guideline development group members have been recorded and addressed	23		Change in underline part
Overall guideline assessment			Rate the overall quality of this guideline	1	Overall guideline assessment	New item
	1	I would recommend this guideline for use	I would recommend this guideline for use	2		Renumber to 2

As there was no item in AGREE I that corresponded with the new overall guideline assessment item in AGREE II, we attempted to calculate this value using two approaches. First, we calculated the average of the standardized score using results of the six AGREE I domains. Second, we calculated the standardized score using the results of the 23 AGREE I items. We examined the correlation between “Overall Guideline Assessment” in the AGREE II and the results of the two approaches described above.

We used t-tests to compare standardized scores, and calculated correlation coefficients for each AGREE I and AGREE II item and domain. p values < 0.05 were indicated statistical significance. All analyses were performed using SPSS, version 20.0 (IBM SPSS Statistics for Windows, Version 20.0. Armonk, NY: IBM Corp.).

### Results

**Fig. 1:**
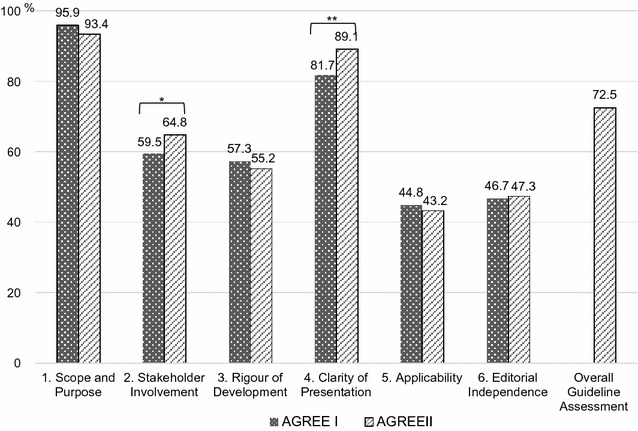
Evaluation of clinical practice guidelines, published between 2011 and 2012, using the AGREE I and AGREE II (n = 68). t test; *p < 0.05, **p < 0.01

The results of the AGREE I and AGREE II evaluations are shown in Fig. 1. Correlation coefficients are shown in Table 2. High correlations were observed in all domains: scope and purpose = 0.758; stakeholder involvement = 0.756; rigor of development = 0.992; clarity of presentation = 0.865; applicability = 0.938; and editorial independence = 0.938. The correlation coefficients of each item ranged from 0.708 to 0.982.

**Table 2:** Correlation between the AGREE I and AGREE II domains

	AGREE I								AGREE II							
	1	2	3	4	5	6	Total	Average	1	2	3	4	5	6	Total	Overall guideline assessment
AGREE I																
1. Scope and purpose	1.000	0.144	0.366**	0.162	− 0.077	0.172	0.371**	0.332**	0.758**	0.197	0.365**	0.100	− 0.051	0.156	0.368**	0.328**
2. Stakeholder involvement		1.000	0.121	0.352**	0.106	− 0.059	0.302*	0.308*	0.113	0.708**	0.107	0.229	0.248*	− 0.079	0.233	0.114
3. Rigour of development			1.000	0.428**	0.243*	0.491**	0.938**	0.849**	0.260*	0.013	0.982**	0.483**	0.263*	0.491**	0.943**	0.736**
4. Clarity of presentation				1.000	0.122	0.069	0.524**	0.502**	0.243*	0.337**	0.397**	0.702**	0.333**	0.106	0.497**	0.339**
5. Applicability					1.000	0.146	0.423**	0.505**	− 0.042	0.007	0.171	0.106	0.919**	0.141	0.361**	0.011
6. Editorial independence						1.000	0.561**	0.647**	− 0.008	0.167	0.484**	0.079	0.181	0.971**	0.575**	0.464**
Total							1.000	0.968**	0.268*	0.278*	0.905**	0.524**	0.473**	0.561**	0.978**	0.685**
Average								1.000	0.211	0.302*	0.810**	0.468**	0.552**	0.646**	0.937**	0.628**
AGREE II																
1. Scope and purpose									1.000	0.174	0.276*	0.251*	− 0.004	0.001	0.303*	0.183
2. Stakeholder involvement										1.000	0.161	0.289**	0.129	0.159	0.316**	0.247*
3. Rigour of development											1.000	0.501**	0.182	0.479**	0.938**	0.771**
4. Clarity of presentation												1.000	0.155	0.092	0.570**	0.492**
5. Applicability													1.000	0.189	0.403**	0.033
6. Editorial independence														1.000	0.575**	0.445**
Total															1.000	0.746**
Overall guideline assessment																1.000

* p < 0.05, ** p < 0.01

Correlation coefficients for the 22 items ranged from 0.694 to 0.995; 16 items had a correlation coefficient of 0.9 or more, three items were 0.8–0.9, and three items were 0.6–0.8. A high overall correlation was observed for all items (Additional file 1: Table S1).

The newly-introduced overall assessment item “Overall Guideline Assessment” (AGREE II) should be assessed based on AGREE I data. The six AGREE I domains had a correlation coefficient of 0.628, when 23 items were used it was 0.685, suggesting a higher related value could be gained using the latter (Table 2).

### Discussion

Since its publication in 2003, the high popularity of the AGREE instrument has produced a large amount of evaluation data. With the revision of the AGREE instrument, the relationship between data obtained from AGREE I and AGREE II, and data conversion from the AGREE I to the AGREE II are high research agenda priorities for investigating time trend analyses of CPG quality.

For the 68 CPGs issued in 2011–2012, our results demonstrated that AGREE I and AGREE II were highly correlated at both the domain and item levels, and the newly introduced overall rating item “Overall Guideline Assessment” could be calculated more precisely using the 23 AGREE I items, rather than domain-level data.

Increasing attention is being directed to safety and quality issues, and CPGs based on EBM are a representative method for standardizing and improving the quality and safety of healthcare procedures. The AGREE instrument is widely used to measure CPG quality. Our results suggest that the AGREE instrument can still be used as a measurement tool, which exhibits high consistency, although it has now been revised (AGREE II). It enables long-term, comprehensive CPG evaluation. The Japanese government has promoted CPG preparation since 1996. Our study may help evaluate the underlying policy guidelines.

### Conclusion

Data obtained from AGREE I can be transferred to the AGREE II, and the data for “Overall Guideline Assessment” can be calculated with high reliability using a standardized score of the 23 items.

## Limitations

Our evaluation team did not include any researchers or clinicians. However, the expert librarians had extensive knowledge about CPG preparation and had experience evaluating CPGs using the AGREE measure.

## Additional file

**Additional file 1: Table S1.** Correlation between the AGREE I and AGREE II items. *p < 0.05, **p < 0.01.

## Abbreviations

AGREE: Appraisal of Guidelines for Research and Evaluation
CPGs: clinical practice guidelines
EBM: evidence-based medicine
